# Supplementary material for: Molecular Characterization of Hepatitis B Virus in People Living with HIV in Rural and Peri-Urban Communities in Botswana
Source: Biomedicines. 2024 Jul 14;12(7):1561. doi: 10.3390/biomedicines12071561 (PMC11275055; doi:10.3390/biomedicines12071561)
Supplement: Supplementary file 1 [file biomedicines-12-01561-s001.zip › biomedicines-3081666-supplementary.pdf]

Table S1. HBV primers used for sequencing PCR

Primer Name	Sequence	Positions
SC_1_LEFT	TTCCACCAAGCTCTGCAAGATC	11 - 32
SC_1_RIGHT	AGAGGAATATGATAAAACGCCGCA	384-407
SC_2_LEFT	CTCCAATCACTCACCAACCTCC	325-346
SC_2_RIGHT	AAAGCCCTACGAACCACTGAAC	692-713
SC_3_LEFT	AAATACCTATGGGAGTGGGCCT	632-653
SC_3_RIGHT	TTGTGTAAATGGAGCGGCAAAG	1655-1676
SC_4_LEFT	AGAAAACTTCCTGTTAACAGACCTATTG	949-976
SC_4_RIGHT	GGACGACAGAATTATCAGTCCCG	1326-1348
SC_5_LEFT	TCCATACTGCGGAACTCCTAGC	1265-1286
SC_5_RIGHT	TGTAAGACCTTGGGCAGGATTTG	1632-1654
SC_6_LEFT	CTTCTCATCTGCCGGTCCGTGT	1559-1580
SC_6_RIGHT	AGAAGTCAGAAGGCAAAAACGAGA	1947-1970
SC_7_LEFT	GGCTTTGGGGCATGGACATT	1890-1909
SC_7_RIGHT	ATCCACACTCCGAAAGAGACCA	2256-2277
SC_8_LEFT	GACAACTATTGTGGTTTCATATTTCT	2193-2218
SC_8_RIGHT	TTGTTGACACCTATTAATAATGTCCTCA	2576-2594
SC_9_LEFT	TGGGCTTTATTCCTCTACTGTCCC	2492-2515
SC_9_RIGHT	GGGAACAGAAAGATTCGTCCCC	2889-2910
SC_10_LEFT	TTGCGGGTCACCATATTCTTGG	2816-2837
SC_10_RIGHT	GGCCTGAGGATGACTGTCTCTT	3189-3210

Primers adopted from Choga et al 2023 [18] (https://dx.doi.org/10.17504/protocols.io.5qpvo3xxzv4o/v1)

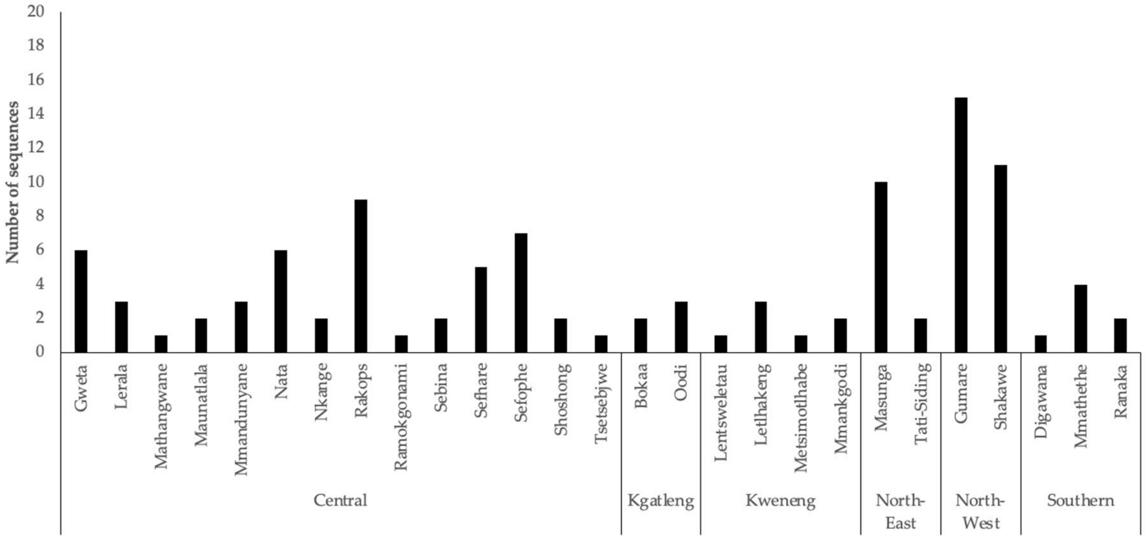

Figure S1. Number of sequences generated per BCPP communities in the different Botswana districts

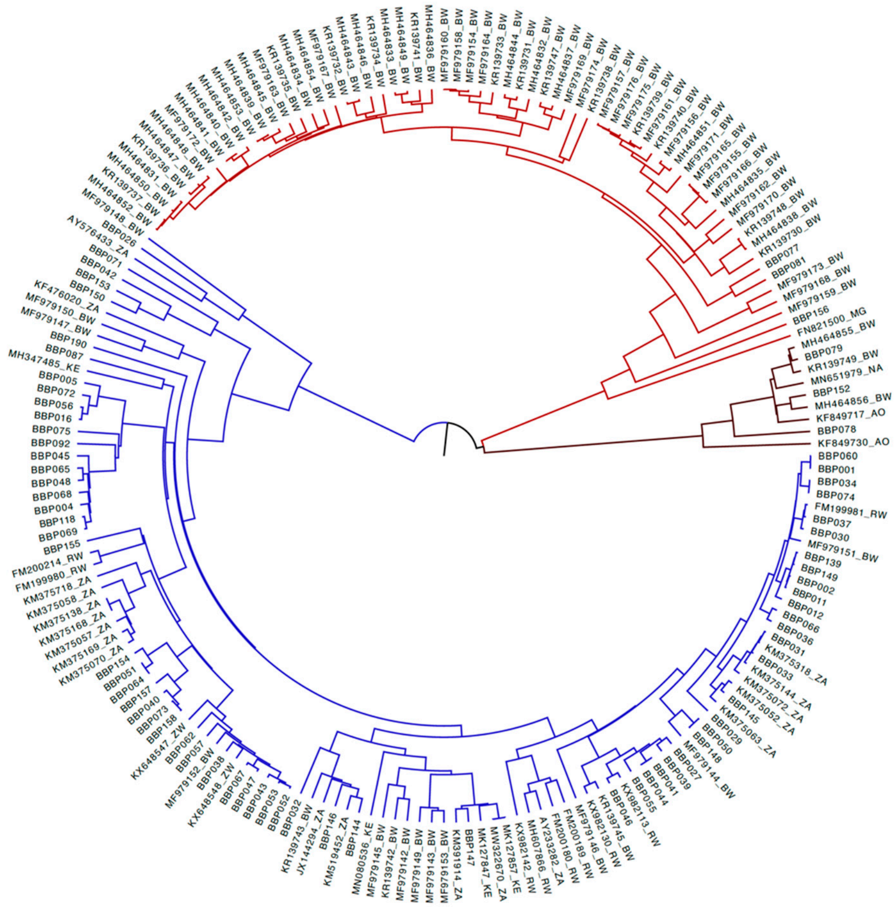

Figure S2: Bayesian phylogenetic tree of sequences generated in the BCPP cohort and reference sequences. Study sequences are denoted by BBP followed by a number while references sequences start with accession number followed by country. Sequences in blue are genotype A1, those in red are genotype D3 and those in black are E.

Table S2. Characteristics of participants with escape mutations

Mutation	Participant	Community	District	Art status	ART regimen	Viral load (IU/mL)	HBV Type
**T114S**	BBP061	Shoshong	Central	On ARVs	ABC + 3TC + LPV	TND	HBsAg+
	BBP058	Gumare	North West	On ARVs	FTC + LPV + TDF	<20	HBsAg+
**S114L**	BBP089	Masunga	North East	On ARVs	DTG+NVP	TND	HBsAg+
**T118M**	BBP083	Rakops	Central	On ARVs	Not available	26	OBI
**C121R**	BBP171	Metsimotlhabe	Kweneng	On ARVs	DTG + TDF	<20	OBI
**K122R**	BBP004	Gweta	North West	On ARVs	EFV + FTC + TDF	11837	HBsAg+
	BBP045	Masunga	North East	On ARVs	Not available	TND	HBsAg+
	BBP048	Mmathethe	Southern	On ARVs	EFV + FTC + TDF	1252	HBsAg+
	BBP049	Gumare	North West	On ARVs	EFV + FTC + TDF	39177	HBsAg+
	BBP061	Shoshong	Central	On ARVs	ABC + 3TC + LPV	TND	HBsAg+
	BBP065	Masunga	North East	On ARVs	Not available	TND	HBsAg+
	BBP068	Gumare	North West	On ARVs	3TC + NVP + ZDV	108	HBsAg+
	BBP069	Masunga	North East	On ARVs	3TC + NVP + ZDV	>170000000	HBsAg+
	BBP118	Gweta	North West	On ARVs	3TC + NVP + ZDV	TND	HBsAg+
**Q129C**	BBP171	Metsimotlhabe	Kweneng	On ARVs	DTG + TDF	<20	OBI
**G130C**	BBP171	Metsimotlhabe	Kweneng	On ARVs	DTG + TDF	<20	OBI
**N131T**	BBP061	Shoshong	Central	On ARVs	ABC + 3TC + LPV	TND	HBsAg+
	BBP146	Sefhare	Central	On ARVs	Not available	1296	HBsAg+
**T131N**	BBP156	Gumare	North West	On ARVs	FTC + NVP + TDF	<20	HBsAg+
**C137I**	BBP085	Rakops	Central	On ARVs	3TC + ZDV	21	HBsAg+
**C139R**	BBP010	Mmankgodi	Kweneng	On ARVs	EFV + FTC + TDF	20	HBsAg+
**N146S**	BBP170	Lerala	Central	On ARVs	EFV + FTC + TDF	<20	OBI
**C147Y**	BBP085	Rakops	Central	On ARVs	3TC + ZDV	21	HBsAg+

Table S3. List of mutations in HBV ORFs

	REFERENCE SEQUENCES			BCPP SEQUENCES			COMMON MUTATIONS		
	**Mutation**	**Frequency**	**Prevalence**	**Mutation**	**Frequency**	**Prevalence**		**REF**	**BCPP**
PreS1	S5P	3	8.1%	G35R	12	12.1%	T94P	44.4%	50.5%
	A6S	6	16.2%	N37Y	15	15.2%			
	K10N	3	8.1%	I48V	21	21.2%			
	F25L	5	13.5%	A90V	23	23.2%			
	V74F	2	5.4%						
	I84V	5	13.5%						
	A86T	2	5.4%						
	V88L	3	8.1%						
	A90T	6	16.2%						
	A90P	9	24.3%						
	V91S	5	13.5%						
	P92L	4	11.1%						
	P92S	5	13.9%						
	P93S	4	11.1%						
PreS2	V17I	2	5.3%	Y21C	2	2.2%	A7T	5.3%	16.9%
	Y21S	2	5.1%	L32V	8	9.0%	A11T	5.3%	10.3%
	F22L	3	7.7%	N37T	7	7.9%	T38I	41.0%	30.3%
	R48T	5	12.8%	A39V	9	10.1%	A53V	12.8%	4.5%
				H41P	2	2.2%	L54P	15.4%	14.6%
				H41L	3	3.4%			
				I45T	18	20.2%			
				S46F	8	9.0%			
				S47L	2	2.2%			
				T49I	6	6.7%			

				D51V	6	6.7%			
				L54S	21	23.6%			
preC				C14Y	2	7.14%	None		
				W28L	3	10.71%			
				V17F	15	53.57%			
Core	S35A	2	8.3%	P45S	8	9.8%	None		
	A41S	2	8.3%	C61R	2	2.4%			
	C48G	2	8.3%	G63R	2	2.4%			
	E64D	3	12.5%	E64K	3	3.7%			
	L65V	3	12.5%	M66T	2	2.4%			
	T67N	4	16.7%	N74I	3	3.7%			
	N74S	2	8.3%	S106F	2	2.4%			
	E77D	2	8.3%	T142S	2	2.7%			
	A131P	3	12.5%	V148A	2	2.7%			
	R151C	4	16.7%	R181P	3	3.7%			
	D153A	3	12.5%						
	S183P	2	8.3%						
X	E80A	2	8.3%	L5V	16	26.7%	S11P	12.5%	18.3%
	I88S	2	8.3%	Y6C	9	15.0%	G22S	8.3%	43.8%
	K130M	3	15.8%	S12A	11	18.3%	A31T	8.3%	28.1%
	V131I	4	21.1%	R26C	13	20.3%	S46P	41.7%	67.2%
				P29S	17	26.6%			
				A31S	8	12.5%			
				G32R	8	12.5%			
				P33S	27	42.2%			
				T36G	3	4.7%			
				T36A	14	21.9%			
				S47A	5	7.8%			

				D48V	6	9.4%			
				C78R	8	12.7%			
				A85S	2	3.4%			
				L93S	2	4.3%			
				V116L	2	4.3%			
				S147P	2	4.3%			
Surface	M1R	2	5.4%	N3S	3	3.5%	T114S	3.4%	2.2%
	S45P	3	8.1%	G7R	3	3.5%	K122R	2.3%	10.1%
	L49R	2	5.4%	F8L	2	2.3%	N131T	14.8%	2.2%
	L98V	3	3.4%	R24K	4	4.5%	V194A	37.5%	5.7%
	M103I	3	3.4%	G44S	2	2.3%			
	P111A	3	3.4%	S45T	2	2.3%			
	S117R	2	2.3%	S45A	4	4.5%			
	Q129R	4	4.5%	P46T	3	3.4%			
	G130N	2	2.3%	S55P	9	10.2%			
	T140I	2	2.3%	T57I	5	5.6%			
	A159V	5	5.7%	V96A	9	10.1%			
	E164G	4	4.5%	Q101K	2	2.2%			
	V168A	2	2.3%	W156Q	2	2.3%			
	L173P	3	3.4%	A159I	2	2.3%			
	V184A	4	4.5%	E164D	25	28.7%			
	S204N	8	9.1%	F179L	2	2.3%			
				V180A	6	6.9%			
				W182R	2	2.3%			
				F183S	3	3.5%			
				V184L	2	2.7%			
				G185W	2	2.7%			
				I195M	39	55.7%			

		W199R	2	2.9%	
		Y200C	2	2.9%	
		P203Q	2	2.9%	
		Y206H	2	2.9%	
		Y206R	3	4.3%	
		I213M	2	2.9%	
		P214L	4	5.7%	

Table S4. List of mutations in HBV polymerase domains

	REFERENCE SEQUENCES			BCPP SEQUENCES			COMMON MUTATIONS		
	**Mutation**	**Frequency**	**Prevalence**	**Mutation**	**Frequency**	**Prevalence**		**REF**	**BCPP**
TP	D16A	3	7.7%	Q6H	2	2.8%	V71I	28.2%	34.7%
	T18L	3	7.7%	A20V	2	2.8%	Q87H	53.8%	47.2%
	V71N	4	10.3%	A20G	2	2.8%	H182Q	5.1%	21.7%
	E75D	4	10.3%	L23P	2	2.8%			
	N120K	2	5.1%	D32G	2	2.8%			
	S121G	2	5.1%	A40I	2	2.8%			
	K161Q	2	5.1%	G46Q	2	2.8%			
	L181I	3	7.7%	W54L	2	2.8%			
				F61L	2	2.8%			
				S80P	2	2.8%			
				P82H	2	2.8%			
				E88R	2	2.8%			
				I91S	13	18.1%			
				R93K	2	2.8%			
				Q95E	2	2.8%			

				M113I	2	2.8%			
				D128N	2	2.8%			
				Q138H	15	20.8%			
				L153T	2	3.8%			
				G157R	2	3.8%			
				R162K	2	3.8%			
				S167G	2	4.3%			
Spacer	V4A	3	7.9%	S15P	7	11.9%	P64A	44.7%	36.3%
	S5I	6	15.8%	S18P	12	20.3%	I84T	13.2%	12.1%
	Q6K	15	39.5%	V29I	6	6.7%	Y86H	13.2%	26.4%
	A7T	23	60.5%	R34Q	11	12.2%	H93S	44.7%	45.1%
	R10W	3	7.9%	Q36L	15	16.7%	S125N	5.6%	22.9%
	Q19K	5	13.2%	H47R	19	20.9%	S129N	5.3%	12.3%
	P20S	2	5.3%	R63W	2	2.2%			
	L24P	5	13.2%	F74L	2	2.2%			
	V29T	3	7.9%	S91A	11	12.1%			
	P46S	3	7.9%	H93P	2	2.2%			
	P56S	4	10.5%	K102E	3	3.3%			
	H83R	5	13.2%	F126I	4	8.2%			
	G85D	2	5.3%	P127S	12	23.1%			
	S87I	2	5.3%	P128S	5	9.6%			
	S89N	6	15.8%	S130F	2	3.2%			
	S89T	9	23.7%	A131S	9	12.7%			
	S90K	5	13.2%	Q136E	2	2.5%			
	S91F	5	13.2%	Q136K	5	6.2%			
	S92L	3	7.9%	G137R	12	14.5%			
	L108F	3	8.1%	P138L	2	2.4%			
	V121M	2	5.4%	S141P	3	3.6%			

	F123L	2	5.6%	T150I	2	2.2%			
	S129G	2	5.4%	T150S	7	7.9%			
	S135N	2	5.4%	Q151E	5	5.6%			
	F140S	4	10.5%	K155D	6	6.7%			
	L158I	10	26.3%	S159T	4	4.5%			
	L161I	2	5.3%						
RT	E1D	5	12.5%	V7T	2	2.3%	V7A	40.0%	16.3%
	H9Q	3	7.5%	E11K	3	3.5%	L53I	35.0%	11.4%
	S105T	5	12.5%	R15K	3	3.5%	I103V	37.5%	23.5%
	S106C	3	7.5%	I16T	2	2.3%	P109S	20.0%	14.1%
	R110G	12	30.0%	A21T	2	2.3%	H122N	35.0%	2.4%
	S119C	3	7.5%	R51G	2	2.3%	W153R	7.5%	19.0%
	Q139H	12	30.0%	G52E	2	2.3%	K266V	27.5%	14.5%
	R242K	3	7.5%	L53N	2	2.3%	K266I	65.0%	65.2%
	H271P	2	5.0%	L53S	2	2.3%	N332S	39.3%	15.9%
	H271S	3	7.5%	T54Y	2	2.3%	Q333K	44.4%	14.5%
	H271C	13	32.5%	T54S	4	4.5%			
	T322I	3	9.1%	V63A	7	8.0%			
				H122L	2	2.4%			
				N124H	19	22.4%			
				Y126H	20	23.5%			
				L129M	2	2.4%			
				N131D	16	18.8%			
				Y151F	2	2.4%			
				R167H	2	2.4%			
				V173L	23	27.4%			
				L180M	41	47.1%			
				I187A	2	2.3%			

				V190A	2	2.3%			
				R192S	2	2.8%			
				R192L	3	4.2%			
				R193V	2	2.8%			
				M204V	37	53.6%			
				V207A	2	2.9%			
				V214A	2	2.9%			
				T222A	2	2.9%			
				A223V	2	2.9%			
				T225A	2	2.9%			
				L229F	2	2.9%			
				N238T	9	13.0%			
				S246F	3	4.3%			
				M250L	4	5.8%			
				V253I	10	14.5%			
				S256G	6	8.7%			
				T259S	8	11.6%			
				Q262R	2	2.9%			
				I282V	8	11.6%			
				C287Y	2	2.9%			
				R289E	2	2.9%			
				R289K	2	2.9%			
				I290F	2	2.9%			
				Y312H	2	2.9%			
				M336V	2	2.9%			
				Y339C	2	2.9%			
				Y339L	2	2.9%			
RNaseH	V138D	3	12.50%	R1Q	2	9.5%	S2P	41.7%	61.9%

		C5M	2	9.5%	Y116F	8.3%	30.0%
		T37A	2	9.1%	R151K	8.3%	7.8%
		A38T	2	9.1%			
		K53N	2	4.1%			
		L54I	2	3.7%			
		G75S	3	4.7%			
		T77A	10	15.6%			
		G84R	2	3.1%			
		A93V	2	2.9%			
		P100L	2	2.9%			
		L107V	4	5.7%			
		Y108S	3	4.3%			
		R113S	2	2.9%			
		L114P	2	2.9%			
		P115L	5	7.1%			
		R117H	2	2.9%			
		R117C	2	2.9%			
		R117Q	6	8.6%			
		T119S	8	11.4%			
		V128D	18	25.7%			
		F142S	3	4.3%			
		V148A	24	36.9%			
